# Factors influencing the maternal choice of packaged snacks for 6–10 years old children: A cross-sectional study from Sri Lanka

**DOI:** 10.1371/journal.pgph.0003574

**Published:** 2024-09-04

**Authors:** Dulshani Pujitha Gunawardhana, Ishanka Ayeshwari Talagala

**Affiliations:** 1 Post Graduate Institute of Medicine, University of Colombo, Borella, Sri Lanka; 2 Department of Community Medicine and Family Medicine, Faculty of Medicine, University of Moratuwa, Moratuwa, Sri Lanka; Al-Bayan University, IRAQ

## Abstract

Childhood overweight and obesity due to unhealthy diet result in several adverse effects. Mothers play a major role in selecting snacks for younger children. This study assessed the factors associated with the choice of packaged food/beverage snacks among mothers of 6- to 10-year-old children in the Medical Officer of Health area Balangoda. A community-based descriptive cross-sectional study with an analytical component was conducted in 2022 among 450 mothers with 6- to 10-year-old children through two-stage, stratified (year of birth considered as a stratum), simple random sampling technique in ten randomly selected Public Health Midwife areas in the medical officer of health area Balangoda. An interviewer-administered questionnaire was used to assess participants’ sociodemographic, usual practices, attitudes, and knowledge toward packaged snacks. Factors associated with maternal practices related to packaged snack selection were assessed through the chi-square test at the p<0.05 significance level. The response rate was 99.3% (N = 447). Children were commonly provided with biscuits (94.4%) and flavoured milk (44.7%) daily (66.4%) at home (93.5%). The majority of mothers reported poor practices in selecting packaged snacks (88.8%), but good knowledge (62.9%) and favorable attitudes (93.5%) toward packaged snacks. The majority (75.5%) were aware that snacks are important for overcoming the hunger gap among children. Childs’ preference (77.6%) was the leading influencing factor while, the nutritional value of the packaged snacks minimally influenced the maternal (2.7%) choice. Only 49.9% mothers knew that packaged fruits could be consumed as snacks. Maternal Sinhalese ethnicity (p<0.001), secondary or higher education (p<0.001), having one child (p = 0.003), residing in a rural or urban area (p = 0.011) and having favourable attitudes (p = 0.002) were significantly associated with poor maternal practices in packaged snack selection. Although mothers’ knowledge toward packaged snacks were good, their practices in selecting packaged snacks were poor. Maternal ethnicity, educational level, number of children, area of residence and attitudes were significantly associated with mothers’ selection of packaged snacks. The results of the study highlighted the need to plan, develop and implement focused health promotion programmes to empower mothers to select healthier packaged snack options for their 6- to 10-year-old children.

## Background

Childhood overweight and obesity result in psychological comorbidities in the short term and chronic noncommunicable diseases (NCD) in the long term [1]. These debilitating consequences affect individuals, societies, and countries [2], making them important public health issues worldwide, especially in lower- and middle-income countries. In addition, overweight and obesity acquired during childhood have been shown to persist into adulthood with low availability of effective treatment modalities [3], highlighting the importance of preventing childhood overweight and obesity. The prevalence of childhood overweight and obesity is reportedly rapidly increasing in most high-income countries as well as in low- and middle-income countries, including Sri Lanka, an island in the South Asia region, during the past three decades [4]. Reportedly, there is a 1.5-fold increase in the global prevalence of childhood obesity in the time between 2012 to 2023, compared to that of 2000–2011 [5].

Although the prevalence of childhood overweight and obesity is lower in Sri Lanka than in other regions, it has shown an increasing trend over the years. Evidently, the prevalence of overweight and obesity in school children (6–12 years) in 2003 were 4.4% and 1.8% respectively, which had increased up to 6.1% and 2.9% respectively by the year 2016 [6]. As per the national nutrition and micronutrient survey the overweight and obesity among 5–9 years children were 4.1% and 2.6% respectively [7]. Furthermore, there are regional disparities, indicating an increasing trend of childhood overweight and obesity within suburban/rural areas of the country [8, 9]. Childhood overweight and obesity negatively impact physical, psychological, and social wellbeing and thus the overall quality of life of these children [10, 11]. Therefore, prompt action is needed towards its prevention and control. Diet-related NCDs later in life are reportedly a result of unhealthy dietary practices that are acquired during childhood, indicating the importance of inculcating healthy dietary practices during childhood and maintained during adolescence [12]. Hence, all efforts should be undertaken to reshape the dietary practices of children and encourage them to adopt healthier options.

In addition to the main meals, snacks play an important role in the diet of children. Snacking is defined as a small amount of food, drink, or light meals eaten between main meals [11], and snacking is essentially important for filling the hunger gaps among children due to their growth spurt. However, snacking among children can be influenced by factors such as taste, convenience, preference, affordability, availability, peer pressure, social desirability of certain food and media influence [13, 14]. The wide availability and promotion of affordable unhealthy snack food [14] had made the matter worse. Further, it is reportedly emphasized that compared to the developed countries, the availability of healthy snack food is scarce in developing countries like Sri Lanka, rather the availability of unhealthy snacks including processed food was higher in low-income countries [4]. Thus, considering the remarkable increase in the prevalence of snacking among children over the years, it is imperative to consider the type of snack, the nutritional quality of the snack and the amount of energy provided by snacking food and beverages.

With the evolution of the food industry, there are plenty of packaged snacks in the market. It was clear that healthy options were seen as limited while fast snack foods were widely accessible [15]. Snacking food and beverages containing high amounts of sugar increases the caloric intake, leading to increased body weight among children. In addition, snacking between main meals in a nonhungry state results in excess weight gain [16]. Since the frequency of snacking was also found to be positively associated with high energy intake in children [17], it is important that children consume relatively healthy food and drink snack items to combat childhood obesity and its adverse consequences.

It was reported that, of all food providers, mothers play an important and responsible role, especially when the children are young [18]. It is well known that mothers usually provide commercially prepared packaged food and beverages to their children as snacks [19]. Reportedly, wide availability and relatively cheap price influences the maternal decision on packaged snack selection for their children [20, 21]. Fielding-Singh, 2017 revealed that most mothers struggled to make the best packaged snacking food/drink choice for their children [13]. Limited knowledge on food based dietary guidelines and recommendations, marketing strategies and reading food labels are few leading factors that explain the poor maternal choice of packaged food/beverage snacks [22, 23]. On the other hand, having a female child, maternal high educational level, healthy lifestyle among mothers including intake of high-quality snacks and utilization of healthy feeding practices were found to be associated with high recognition level of dietary recommendations [22] and in promoting healthy packaged snacking behavior among children [24, 25].

In the mother-centered family context in Sri Lanka, she is the meal planner for her family, including snacks for her children. Further, children in the six-to-ten years age group who are in school grades one to five usually have low purchasing power and autonomy and are more dependent on their mothers for their snacking. Therefore, it is essential that mothers have clear, good knowledge and favourable attitudes toward packaged food/drink snacks and are empowered to choose healthy snacks for their children.

Hence, mothers’ choice of healthy packaged snacks for their children aged 6–10 years is an investment in preventing childhood overweight and obesity and thereby chronic NCDs later in life and premature deaths. Thus, the current study was conducted with the objective of obtaining insight into the factors associated with the choice of packaged food/beverage snacks for children among mothers of 6- to 10-year-old children in the Medical Officer of Health (MOH) area Balangoda (the MOH area is the basic regional-level preventive healthcare delivery administrative unit in Sri Lanka).

## Methods

A community-based descriptive cross-sectional study with an analytical component was conducted among mothers of 6–10-year-old children living in the MOH area Balangoda. Ethical approval was obtained from the Ethical Review Committee, Post Graduate Institute of Medicine, University of Colombo, Sri Lanka. The population of this suburban MOH area has various socioeconomic, racial, and religious backgrounds. Mothers of 6-to10-year-old children who were living in the MOH area Balangoda for more than one year and mothers of 6- to 10-year-old children whose birth was registered in the birth and immunization registry of the area Public Health Midwife (PHM), which is the field-level maternal and child healthcare service delivery subunit within an MOH area, were included in the study. Generally, in the Sri Lankan preventive health system, almost all births are registered in the birth and immunization registry, as children obtain community-based immunization (national immunization programme) through the area PHM [26, 27].

The following were excluded from the study: mothers of 6- to 10-year-old children who were diagnosed with any chronic illness [confirmed by medical records (diagnosis card or clinic book)], as their usual dietary behaviour may differ from that of other children; mothers, whose children generally live away from them for more than three months per year (e.g., children living in school hostels, boarding places, separated/divorced/widowed mothers who are not living with their children); mothers with uncontrolled psychological disorders [confirmed by medical records (diagnosis card or clinic book)] as they are unable to report accurate responses independently.

The required sample size for the study was calculated based on the formula, N = Z^2^ p (1 –p) / d^2^ [28]. The proportion of children aged eight years or younger who snacked along with an adult (44%) [29] was considered as the estimated prevalence, 0.05 precision level and p<0.05 significance level were considered for the sample size calculation. With a 10% expected nonresponse rate, the total required sample size (N) was 421.

A two-stage, stratified, simple random sampling technique was used to select the eligible mothers from 10 randomly selected PHM areas, out of a total of 23 PHM areas in the Balangoda MOH division (first stage). PHM area is the basic field-level public health service (mainly maternal and child health services) delivery unit within the Sri Lankan preventive health system, where the relevant PHM is expected to register all the children born within her care-area according to the year of birth, including relevant details of the parents as well [27]. For equal representation of mothers of 6- to 10-year-old children from the randomly selected PHM areas, 42 mothers (421/10) from each area were to be selected. Since there were five (05) birth cohorts (birth cohort considered as a stratum) under concern (2012, 2013, 2014, 2015 and 2016), nine (09) children (42/5 = 8.4) from each birth cohort were recruited into the study, allowing equal representation from each birth cohort in the sample. Thus, from each PHM area, a total of 45 children were selected randomly (second stage), based on the eligibility criteria, from the birth and immunization register of the relevant PHM using computer-generated random numbers. Thus, 450 mothers of 6- to 10-year-old children were ultimately randomly recruited for the study. After obtaining administrative clearance from the Regional Director of Health Services, Rathanpura District, data collection was started from 21/09/2022 and completed within four weeks. Following obtaining informed written consent from each study participant, data were collected on appointment basis, at the study participant’s home or at a convenient place selected by her.

An interviewer administered questionnaire gathered information on participants’ sociodemographic, economic and health characteristics; usual practices related to selecting packaged food/beverage snacks for their children; and knowledge and attitudes on packaged food/beverage snacks. The questionnaire was primarily developed by the investigators (based on literature and following discussions with a panel of experts) in English language and was translated into local languages (Sinhala and Tamil). Backward translation was also done to English language to assess its accuracy. Judgmental validity (face validity and content validity) was assessed by a panel of experts consisted of Specialist in public health, paediatrics and nutritionists e. Pre-testing of the questionnaire was done among twenty mothers of 6-10-years old children in the adjacent medical officer of health area–Imbulpe. The data collectors were well trained during a three-day workshop by the principal investigator prior to the data collection.

Mothers’ practices related to packaged snack choices and their knowledge and attitudes toward packaged food/drink snacks were assessed based on three separate predetermined marking schemes established with the support of the expert panel. Mothers who had scored more than 60% of the total marks were categorized as having ‘good’ practices related to packaged snack choices for their children, while those who obtained lower marks were categorized as having ‘poor’ practices. Similarly, mothers who had a score greater than 67% were considered as those with ‘good’ knowledge of packaged snacks, and those with a score greater than 62% were considered as those with ‘favorable attitudes’ toward packaged snacks, based on the respective marking schemes.

Descriptive statistics, means (SDs) and proportions for numerical and categorical data respectively were used. Factors associated with the mothers’ choice of packaged snacks were assessed using chi-square test, at p<0.05 significance level, using SPSS 26^th^ version.

## Results

Out of 450 participants, only 99.3% responded (N = 447). The mean age of the participants was 33 years (SD = 4.5). Most of the mothers were Sinhalese (68.9%), Buddhist (68.2%), from urban areas of the MOH division (70.2%), married (98.9%), having one child (80.1%) between 6 and 10 years of age, with a secondary or higher education (90.4%), ever employed (66.7%) and with a low income (LKR ≤50,000; 61.7%). The demographic and socio-economic characteristics of the participants are shown in Table 1.

**Table 1:** Sociodemographic, economic, and clinical characteristics of the mothers.

Socio-Demographic, economic and clinical characteristic	Frequency	
	Number (N = 447)	Percentage (%)
**Maternal age**		
< 33 years	246	55.0
> 33 years	201	45.0
**Ethnicity**		
Sinhala	308	68.9
Tamil	93	20.8
Muslim	46	10.3
**Religion**		
Buddhist	305	68.2
Hindu	87	9.5
Islam	46	10.3
Catholic	9	2.0
**Marital state**		
Married	442	98.9
Unmarried	2	0.4
Divorced/Widowed/Separated	3	0.7
**Number of children aged 6–10 years**		
One child	358	80.1
More than one child	89	19.9
**Highest level of education**		
No education or primary only	39	9.6
Secondary or higher	408	90.4
**Occupational status**		
Ever employed	298	66.7
Never employed	149	33.3
**Average monthly family income**		
≤ Rs.50,000.00	276	61.7
> Rs.50,000.00	171	38.3
**Area of residence**		
Urban	314	70.2
Estate	70	15.7
Rural	63	14.1

Of the mothers, only 11% (n = 49) claimed that they were diagnosed with a chronic non-communicable disease. Of them (n = 49), 38.8% were diagnosed with diabetes and 36.7% with hypertension.

### Practices of mothers related to the provision of packaged snacks for their 6- to 10-year-old children

The majority of mothers provided packaged snacks to their 6- to 10-year-old children daily (66.4%), at least once (63.3%) per day, mostly at home (93.5%) (Table 2).

**Table 2:** Usual maternal practices related to provision of packaged food/beverage snacks for their 6- to 10-year-old children.

Usual practice related to provision of packaged snacks for their child/children	Frequency	
	Number (N = 447)	Percentage (%)
**Frequency of provision of packaged snacks per week**		
Daily	297	66.4
2–6 times per week	122	27.3
Less than 2 times per week	28	6.3
**Frequency of provision of packaged snacks per day**		
Once a day	283	63.3
Twice a day	133	29.8
More than thrice a day	31	6.9
**Most common occasion of provision of packaged snacks (N = 447) [**more than one answer was possible]		
At home	418	93.5
Before/after tuition class	149	33.3
During Sunday school interval	116	25.9
During after school extracurricular activities	112	25.1
During school interval	34	7.6

### Usual maternal practice on the choice of packaed food/ beverage snacks for 6-to 10-years-old children’s old children

The maternal selection of the packaged snack was mainly based on the child’s preference (77.6%), while convenience (16%) was also considered. Majority of the mothers had given biscuits (94.4%) and packaged flavoured milk (44.7%) for their 6–10 years old children. Majority of the mothers claimed that they usually read the food label (92.4%) and referred to the expiry date (80.8%), price (80.8%) and the brand name (67.8%) mostly (Table 3).

**Table 3:** Usual maternal practice on the choice of packaged food/beverage snacks for 6- to 10-year-old children.

Characteristic related to maternal practice	Frequency	
	Number(n)	Percentage (%)
**Influencing factors on choice of packaged snacks (N = 447)**		
Child’s preference	347	77.6
Convenience	71	15.9
Nutritional values of packaged food /beverage snacks	12	2.7
Frequency availability of packaged food/ beverage snack	10	2.2
Low price price of the packaged food/ beverage snacks	7	1.6
**Most frequently provided packaged food snack items (N = 447;** More than one answer was possible)		
Biscuits	422	94.4
Yogurt	283	63.3
Chocolate	257	57.5
Instant noodles	228	51.0
Packaged bakery items	225	50.3
Packaged nuts	215	48.1
Packaged ice-cream/ice pops	197	44.1
Cheese	150	33.6
Extruded snacks	137	30.6
Packaged cereals	105	23.5
**Most frequently provided packaged beverage snack item (N = 447)**		
Packaged flavoured milk	200	44.7
Packaged food drinks	195	43.6
Yogurt drink	177	39.6
Fresh milk	147	32.9
Carbonated fizzy drinks	117	26.2
Fresh fruit juice	69	15.4
**Read the food label of packaged snacks (N = 447)**		
Yes	413	92.4
No	34	7.6
**Most referred component of the food label (n = 413;** more than one answer was possible)		
Expiry date	334	80.8
Price	334	80.8
Brand name	280	67.8
Net weight	175	42.3
List of ingredients	115	27.8
Instructions for storage	81	19.6
Colour coding system (traffic light system)	71	17.2
Nutrient panel	64	15.5
Claims	64	15.5
Country of manufacture	24	5.8
Serving size	22	5.3
Contact details of manufacture and distributor	14	3.4
**Overall maternal practice on packaged food/beverage snacks (N = 447)**		
Good practice	50	11.2
Poor practice	397	88.8

However, based on the cut-off score, the majority of the mothers had poor practices (88.8%) in selecting the packaged food or beverage snacks for their 6- to 10-year-old children (Table 3).

### Knowledge of packaged food/beverage snacks

A snack is an additional light meal given between main meals (73.6%), and it is important for young children (86.6%) to fill their hunger gaps (75.5%), were correctly perceived by the mothers. Majority of the mothers correctly knew that packaged snacks could be healthy or unhealthy (95.3%) and could lead to obesity and NCD (91.5%). Alarmingly, nearly half of the participants knew that packaged fruits could be consumed as snacks (49.4%). The majority of mothers correctly identified the nutrient components denoted in the traffic light colour coding in the packaged food snacks (73.6%), and in the packaged beverage snacks (71.8%) and correctly interpreted the ‘Green’ colour coding as healthy to consume (75%). Based on the cut-off used, most mothers had an overall good knowledge (62.9%) on packaged food/beverage snacks (Table 4).

**Table 4:** Maternal knowledge of snacking and packaged food/beverage snacks.

Characteristic of maternal knowledge	Frequency	
	Number (n)	Percentage (%)
**Knowledge on what a snack is (N = 447)**		
Additional light meal given in-between main meal	329	73.6
Easily preparable, instant food	65	14.4
Food/beverage high in sugar, salt, fat and unhealthy	19	4.2
A type of food consumed with tea or plain tea	19	4.2
A type of food prepared outside the home	15	3.6
**Perception on importance of snacking to their children (N = 447)**		
Important	387	86.6
Not important	60	13.4
Importance on snacking to their children (n = 387) *		
To overcome hunger gaps of children	292	75.5
To fulfill previously skipped main meal	48	12.5
Due to previously obtained healthcare advises	41	10.6
Due to media influence	3	0.7
Due to previously obtained information from friends/relatives/neighbors	3	0.7
**Knowledge on packaged food/beverage snacks (N = 447)**		
Packaged snacks could be healthy and/or unhealthy	426	95.3
Packaged snacks may lead to obesity and NCD	409	91.5
Packaged snacks should contain a food label	382	85.5
Packaged snacks may contain low amounts of micronutrient	340	76.1
Packaged fruits could be consumed as snacks	221	49.4
**Identification of the nutrient components of the traffic light colour coding in packaged food Snacks (N = 447)**		
Sugar, salt, fat	329	73.6
Sugar, protein, carbohydrate	58	13.0
Fat, protein, carbohydrate, sugar, salt	47	10.5
Fat, protein, carbohydrate	13	2.9
**Identification of the nutrient components of the traffic light colour coding in packaged beverage snacks (N = 447)**		
Sugar level	321	71.8
Carbohydrate level	54	12.1
Salt level	54	12.1
Fat level	11	2.5
Protein level	7	1.5
**Interpretation of the green color traffic light coding on the food label (N = 447)**		
Healthy to consume	335	75.0
Not know	102	22.8
Unhealthy to consume	10	2.2
**Maternal knowledge category (N = 447)**		
Good knowledge	281	62.9
Poor knowledge	166	37.1

*60 missing data

### Maternal attitudes toward packaged food/beverage snacks

Maternal attitudes differed on several attitude statement areas, as shown in Table 5. However, the overall attitudes toward packaged food/beverage snacks were good for most of the study participants (more than 55%). Nevertheless, 11% of the mothers believed that the consumption of packaged snacks would not affect the health of their children, and 20.6% of them believed that high-energy packaged snacks need to be given to their children as they are active. Among the study participants, 11.2% of the mothers thought that packaged snacks from known brands and packaged snacks familiar to them since childhood (10.5%) were safer. Almost one-third (27.1%) of the mothers believed that milk-based packaged snacks are always healthy (Table 5).

**Table 5:** Attitudes of the study participants toward packaged food/beverage snacks (N = 447).

Attitude statement	Agree		Neutral		Disagree	
	(n)	(%)	(n)	(%)	(n)	(%)
**Children are healthy, consumption of packaged snacks do not affect their health**	49	11	22	4.9	376	84.1
**Known brand names are alright to be given to children**	50	11.2	63	14.1	334	74.7
**Milk based packaged snacks are always healthy**	121	27.1	79	17.7	247	55.2
**Packaged snacks consumed during mothers’ childhood are good for consumption by children**	47	10.5	66	14.8	334	74.7
**Children are active; therefore, high energy snacks need to be given**	92	20.6	61	13.6	294	65.8
**Snack is an additional food/beverage item, it does not need to be healthy**	50	11.2	73	16.3	324	72.5
**Packaged snacks need to be given based on the child’s preference**	46	10.3	101	22.6	300	67.1
**Imported packaged snacks are good in quality**	61	13.6	73	16.3	313	70
**Higher priced packaged snacks are healthier**	52	11.6	40	8.9	355	79.4
**Packaged snacks claimed ‘natural’, are healthier**	90	20.3	70	15.7	286	64
**Snacks with a good quality package are healthier**	56	12.5	58	13	333	74.5
**Packaged snacks frequently advertised by media are healthier**	59	13.2	56	12.5	332	74.3
**Most popular packaged snacks among children are healthier**	38	8.5	76	17	333	74.5

Based on the cut-off used, majority of the mothers (93.9%) had favourable attitudes toward packaged food/beverage snacks (Table 6).

**Table 6:** Categorization of maternal attitudes toward packaged food/beverage snacks.

Attitude category	Frequency	
	Number (n)	Percentage (%)
**Favorable attitude**	420	93.9
**Unfavorable attitude**	27	6.1
**Total**	**447**	**100.0**

### Factors associated with practices related to the choice of packaged food/beverage snacks among mothers

As shown in Table 7, the prevalence of poor practices related to the selection of packaged food or beverage snacks for their 6- to 10-year-old children was greater among Sinhalese mothers (92.5%) than among mothers who were non-Sinhalese (80.6%). This difference in ethnicity among mothers on poor practices of selecting packaged food/beverage snacks was statistically significant (*χ*^2^ = 13.783; df = 1; p<0.0001). Compared to the mothers with a lower educational level (no education or primary education only; 59%), those with a higher educational level (secondary education or higher; 92%) had significantly poor practices related to selecting packaged food/beverage snacks for their 6–10 children (*χ*^2^ = 41.656; df = 1; p<0.0001).

**Table 7:** Factors associated with the choice of packaged food/beverage snacks among mothers.

Demographic, socioeconomic and health-related characteristics	Practices related to the choice of packaged food/beverage snack				Total	Significance
	Good practice		Poor practice		n (%)	
	n	%	n	%		
**Maternal age**						
≤ 33 years	29	11.8	217	88.2	246 (100)	*χ*^*2*^ = 2.000
> 33 years	21	10.4	180	89.6	201 (100)	df = 1
						p = 0.655
**Ethnicity**						
Sinhalese	23	7.5	285	92.5	308 (100)	*χ*^*2*^ **= 13.783**
Non-Sinhalese^1^	27	19.4	112	80.6	139 (100)	**df = 1**
						**p <0.0001**
**Highest Level of education** ^2^						
No education or primary only	16	10.6	23	59.0	39 (100)	*χ*^*2*^ **= 41.655**
Secondary or higher	33	8.1	373	91.9	408 (100)	**df = 1**
						**p<0.0001**
**Number of children in the family**						
One child	38	10.6	320	89.4	358 (100)	*χ*^*2*^ **= 9.954**
More than one child	12	13.5	77	86.5	89 (100)	**df = 1**
						**p = 0.003**
**Employment status**						
Ever employed	31	10.4	267	89.6	298 (100)	*χ*^*2*^ = 0.552
Never employed	19	12.7	130	87.3	149 (100)	df = 1
						p = 0.458
**Monthly family income** ^3^						
≤Rs. 50,000.00	35	9.5	241	90.5	276 (100)	*χ*^*2*^ = 1.624
>Rs. 50,000.00	15	4.4	156	95.6	171 (100)	df = 1
						p = 0.203
**Area of residence**						
Estate	14	20.0	56	80.0	70 (100)	*χ*^*2*^ **= 6.491**
Urban and rural	36	9.5	341	90.5	377 (100)	**df = 1**
						**p = 0.011**
**Diagnosed with a chronic NCD (study participant and/or her spouse)**						
Yes	2	4.1	47	95.9	49 (100)	*χ*^*2*^ = 2.796
No	48	12.1	350	87.9	398 (100)	df = 1
						p = 0.095
**Attitude status**						
Favourable attitudes	42	10.0	387	90.0	420(100)	*χ*^*2*^ **= 9.84**
Un favourable attitudes	8	29.6	19	70.4	27(100)	**df = 1**
						**p = 0.002**
**Level of Knowledge**						
Good knowledge	30	10.7	251	89.3	281(100)	*χ*^*2*^ = 0.198
Poor knowledge	20	12.0	146	88.0	166 (100)	df = 1
						p = 0.657

^1^Tamil and Muslim categories were amalgamated to form the ‘non-sinhalese’ category

^2^ No formal education or only up to primary education categories were amalgamated to form the ‘no education or primary only’ category; and Grade 6–10 only, G.C.E O/L passed, G.C.E A/L passed and certificate/diploma/degree categories were amalgamated to form the ‘secondary or higher’ category

^3^Income < Rs. 20,000 and Rs. 20,001-Rs. 50,000categories were amalgamated to form the ‘<Rs. 50,000.00’ category; and income Rs. 50,001—Rs.100,000 and >Rs. 100,000 categories were amalgamated to form the ‘>Rs. 50,000.00’ category

In addition, poor practices related to the selection of packaged food or beverages for their 6–10 children were significantly (p = 0.003) more common among mothers who had only one child of 6–10 years age group compared to those who had more than one child. Compared to mothers who were residing in the estate sector (80%), a significantly greater proportion of mothers who were residing in urban or rural areas (90.5%) had poor practices related to the selection of packaged snacks (p = 0.011).

Further, although statistically not significant (p>0.05), older mothers (>33years; 89.6%) compared to younger mothers (≤33 years; 88.2%); mothers who were ever employed (89.6%) compared to those who were never employed (87.3%); mothers who had a higher family income (>LKR 50,000) compared to those with a low income (≤LKR 50,000; 90.5%); and those who (or the spouse) were diagnosed with a chronic NCD (96%) compared to those who were not (88%), had poor practices related to selecting packaged food/beverage snacks to their 6–10 year old children.

The majority of mothers who had good knowledge (89.3%) and favourable attitudes (90%) toward packaged snacks had poor practices in selecting packaged food/beverage snacks. However, 88% of mothers with poor knowledge of packaged snacks also had poor practices in selecting packaged food or beverages for their 6- to 10-year-old children. However, the difference observed between the two knowledge categories was not statistically significant (p>0.05). Nevertheless, 70.4% of mothers with unfavourable attitudes toward packaged snacks also had poor practices in selecting packaged snacks. The difference in attitudes between mothers’ attitudes toward packaged snacks and poor practices of selecting packaged food/beverage snacks for their 6- to 10-year-old children was statistically significant (p = 0.002).

## Discussion

The key findings of this study indicated that the majority (62.9%) of the mothers had good knowledge on packaged snacks, and 93.9% of them had favourable attitudes toward packaged snacks. However, only 11.2% of them had good practices related to choosing packaged snacks for their children. In addition, the study revealed that mothers having favourable attitudes toward packaged snacks was negatively associated with practices related to the choice of packaged snacks for their children (p = 0.002).

The current study revealed that the majority of mothers usually provided packaged food or beverages to their 6- to 10-year-old children daily (66%), and many of them had fed their children packaged snacks at least once a day (63%). Similarly, Sharma et al., 2019, in their study using a mixed method, reported that 2/3^rd^ of their study participants (working mothers, nonworking mothers and grandmothers) usually provided commercially prepared packaged food to their children as snacks [20].

In this study, the majority of mothers provided packaged snacks to their children mostly at home (93.5%), before and/or after tuition classes (33%). Since mothers play a major role in meal planning, including snacking, especially among younger children [23], these findings of the present study signify the importance of improving mothers’ knowledge of the provision of healthy snacks to their children. For most of the mothers (78%), the child’s preference was the leading influencing factor for their choice, while the mother’s convenience (16%) was also considered. Similarly, a study conducted among caregivers of children aged 12–23 months in Nepal by using mixed methods (focused group discussions and structured interviewer-administered questionnaires) revealed that adults also provide packaged snacks as a treat for their children [20]. This finding, however, was not reported by the participants of the present study. Our study revealed that biscuits were the most common (94%) packaged snack food item given to their children by the mothers. These findings are similar to those of Talagala and Arambepola (2016), who reported that biscuits were the most frequently snacked food item among Grade 12 students in the Colombo district in Sri Lanka [30]. This could be because of the easy availability and relatively cheaper price of the products.

Yogurt (63%), chocolate (58%), instant noodles (51%), and packaged bakery items (50%) were the other most provided snack food items given by the mothers to their 6- to 10-year-old children, indicating that despite the availability of healthy snack food options, mothers select otherwise. These findings were similar to the findings of a study conducted in the United States, which discovered that the childrens’ most frequent food snack was sweet items including sweet bakery snacks (33.4%), savoury snacks (30.5%) and candies (30%) [31]. This could be due to the limited time and convenience of obtaining these products, especially for working mothers [32], and the relatively low price of these unhealthy snack food items [33].

The current study indicated that the majority (89%) of mothers had poor practices in selecting packaged food/beverage snacks for their 6- to 10-year-old children. Likewise, a qualitative study conducted among 42 mothers and 14 fathers from 44 families revealed that two-thirds of mothers struggled to make the best packaged snacking food/drink choice for their children [13].

Considering that children frequently consume snacks and that their childhood snacking behaviours are maintained throughout adolescence, it is vital that they snack on healthy food/beverage items to prevent adverse health outcomes in the future.

In our study, nearly three-fourths of the mothers (74%) correctly perceived that a ‘snack’ is an additional light meal given between the main meals. This finding is similar to the findings observed in a cross-sectional study conducted in Finland among 25-64-year-old adults, where 84% of the male and 89% of the female participants perceived that a snack is a light meal consumed between main meals [33].

Furthermore, the current study revealed that although the majority (87%) of the mothers of children aged 6–10 years knew that snacking is important for their children, only slightly more than three-fourths of them (76%) correctly knew that snacking is important for overcoming their hunger gaps. A systematic review conducted with the objective of determining the role of snacking in diet quality and body weight also revealed that snacks act as a bridge to adjust and compensate for the energy requirements of children [34].

Among the participants in the present study, 95% of them knew that packaged snacks could be either healthy or unhealthy. On the other hand, only 76% of the participants in the present study knew that packaged snacks contain low amounts of micronutrients, and nearly half (49%) of them knew that packaged fruits can also be considered snacks. However, a cross-sectional study conducted among 30 conveniently sampled children (mean age = 7.1 years; SD = 1 year) in preschools and daycare centers in the U.S. to evaluate the pattern of consumption of packaged fruits and vegetables by children revealed that majority of the children claimed that vegetables and fruits contained in healthy packaging are healthier for them to consume as packaged snacks [35].

Nearly three-fourths of the participants in the present study (74%) could correctly identify the nutrient components that are indicated in the front of pack traffic light color coding in a packaged food snack item as sugar, salt and fat, while only 72% of them correctly identified it in a snack beverage item. Similarly, a cross-sectional study conducted among 200 adults in the Western province of Sri Lanka, with the objective of assessing consumer preference for labeling regulations, reported that 60% of the participants considered traffic light food labeling systems at the point of purchase [36].

However, it is important to note that 11% of the mothers believed that the consumption of packaged snacks does not affect their children’s health, as the children are generally known to be healthy; almost one-third of the mothers believed that high-energy snacks need to be given to the children as they are active (27%); and almost one-third of the mothers (27%) believed that milk-based packaged snacks are always healthy. Similarly, in a study conducted among caregivers of 12-23-month-old children, the majority of the caregivers claimed that packaged food and beverages are unhealthy for consumption by children, whereas milk and milk-based products are healthy for children [20].

Additionally, 10% of the mothers believed that packaged snacks should be selected based on the child’s preference. Furthermore, 9% of the mothers believed that packaged snacks that are popular among children are healthier. These attitudes of the mothers were portrayed in their practices on selecting a packaged snack, where 78% of them selected the snack based on their child’s preference. However, 11% of the participants in the present study believed that packaged food or beverages from known brands are good for consumption by their children. It has been reported that brand shopping is common among adults [37]. worldwide, including in Sri Lanka. Manufacturers and marketers focus on establishing brand preference and brand loyalty among their consumers [14], as brand loyalty often sustains for generations.

In addition, the mothers in the current study believed that food/beverage snack items imported (14%), expensive (12%) and with good quality packaging (13%) are relatively healthier for consumption by their children. Additionally, one-fifth (20%) of the mothers believed that the packaged snack items claimed to be ‘natural’ were healthier and the packaged snack items that are frequently advertised in media to be relatively healthier (13%). These findings were similar to the findings of a study conducted among school-going adolescents in the Colombo district [30]. It is evident that the attitudes borne toward packaged food/beverage snack items during adolescence continue into adulthood and that they act upon their attitudes as adults and transfer them to the future generation as well.

Even though poor practices related to the selection of packaged food/beverage snacks among mothers were mostly observed (90%) among those who were more than 33 years old (the median age of the current study participants) compared to those who were 33 years old or younger (88%), the current study was unsuccessful in identifying a statistically significant difference. With advanced maternal age, usage of print, visual or social media, access to the internet and peer influence may differ, leading to this observed disparity. This finding provides an insight to the policy makers and implementers which specifies the importance of planning and implementing focused educational programmes on packaged snacks for all mothers of 6- to 10-year-old children, irrespective of their age.

The current study revealed that, compared Tamil and Muslim ethnicities, Sinhalese ethnicity of the mothers was significantly associated with poor practices related to the selection of packaged food/drink snacks (p< 0.0001). The presence of different religious backgrounds and cultural beliefs could explain these differences. However, the majority of the mothers in the current study were Sinhalese (69%), which may also be a reason behind this observed difference.

In addition, mothers with a secondary education or higher had poorer practices in selecting packaged food/beverage snacks than mothers with no formal education or mothers educated only up to the primary level (p<0.0001). Similarly, a cross-sectional study conducted among 200 adults in the Western province of Sri Lanka reported that 80.5% of the study participants had a university education and that there was a significant difference in the level of education of the participants on the use of front-of-pack food labels at the point of purchase [8]. Mothers with a higher education level are mostly employed in the current context. Hence, limited time and convenience could be the leading factors that drive mothers in terms of their selection of packaged food or beverage snacks for their children [8], resulting in this observed behaviour. Nevertheless, although poor practices related to packaged food/beverage snack selection among employed mothers were more common (90%) than among never employed mothers (87%), this difference was not statistically significant. This finding is of utmost importance for policy makers and implementers when planning and implementing health education and health promotion programmes for mothers on selecting packaged snacks for their children.

The current study reported that poor practices related to the selection of packaged food/beverage snacks were significantly more common among mothers living in urban or rural areas than among mothers living in real estate areas (p = 0.011). A study conducted in central Malawi, with the aim of assessing the decision to purchase food among Malawian mothers (n = 54) in the dry season, n = 55 in the rainy season), reported a difference in food choices among mothers in urban and rural villages. The authors of the study reported that urban mothers often purchased fruits (30%) more than the rural mothers and that rural mother purchased fried snacks (14%), while urban mothers did not [33]. The differences in cultural norms, dietary practices and behaviors, hygienic practices, economic affordability, and the availability of packaged food/beverage snacks between the estate sector and urban or rural sectors could also explain these findings. However, the current study revealed that poor practices related to the selection of packaged snacks were common (96%) among mothers with higher incomes (>Rs. 50,000) than mothers with a lower income (90.5%), although this difference was not statistically significant.

### Limitations

The participants in the present study were mothers of children aged 6–10 years, living in a sub-urban area considering the general context of the country. Therefore, the generalizability of the current study findings to all mothers and to fathers and caregivers is limited. Mothers of children with chronic diseases, children generally living away from mothers were excluded from the current study, further limiting the generalizability of findings. An interviewer-administered questionnaire was used to collect the data in the current study, compared to a self-administered questionnaire, which would have limited the participants in answering the questions more freely and accurately. Although the data collectors were well trained, there is a possibility of interpreting, extracting and recording the responses differently. The questionnaire assessed the mothers’ usual practices related to the choice of packaged food/beverage snacks for their children, which might have resulted in recall bias as well.

## Conclusion

The findings of the current study highlighted that despite having good knowledge and favourable attitudes toward packaged snacks, the majority of mothers had poor practices with packaged snacks for their 6- to 10-year-old children. Maternal ethnicity, educational level, number of children, area of residence and attitudes towards packaged snacks were significantly associated with the selection of a packaged snack… Therefore, the policy makers should develop, plan and implement focused community-based health promotion programmes aiming at empowering mothers to select healthier packaged snack options for their 6- to 10-year-old children. Implementation of focused community-based health education programmes on reading, understanding and interpreting food labels on packaged snacks are recommended Conducting qualitative studies, such as direct observations and focus group discussions, in order to understand the maternal practices related to selecting packaged food or beverage snacks for their children; reading, interpreting and using food labels; and identifying the factors associated with mothers’ perspectives are recommended.

## Supporting information

S1 Data Data set. (SAV)
